# Association of the Scottish inflammatory prognostic score with treatment-related adverse events and prognosis in esophageal cancer receiving neoadjuvant immunochemotherapy

**DOI:** 10.3389/fimmu.2024.1418286

**Published:** 2024-07-05

**Authors:** Qiang Zhao, Liang Wang, Xun Yang, Jifeng Feng, Qixun Chen

**Affiliations:** ^1^ Department of Thoracic Oncological Surgery, Zhejiang Cancer Hospital, Hangzhou Institute of Medicine (HIM), Chinese Academy of Sciences, Hangzhou, China; ^2^ Key Laboratory of Diagnosis and Treatment Technology on Thoracic Oncology (Lung and Esophagus) of Zhejiang Province, Zhejiang Cancer Hospital, Hangzhou, China

**Keywords:** Scottish inflammatory prognostic score, treatment-related adverse event, esophageal squamous cell carcinoma, neoadjuvant immunochemotherapy, prognosis

## Abstract

**Background:**

To investigate the relationship between the Scottish inflammatory prognostic score (SIPS), treatment-related adverse events (TRAEs), and prognostication in patients with neoadjuvant immunochemotherapy (NICT) for esophageal squamous cell carcinoma (ESCC).

**Methods:**

A retrospective investigation was carried out on 208 ESCC patients treated with NICT. The relationships between the SIPS, TRAEs, and prognosis [disease-free survival (DFS) and overall survival (OS)] were analyzed.

**Results:**

The patients, comprising 62 (29.8%) cases of SIPS0, 103 (49.5%) cases of SIPS1, and 43 (20.7%) cases of SIPS2, were categorized into three groups based on SIPS. Among patients with SIPS2, the oldest age (P=0.006), lowest BMI (P=0.001), longest tumor length (P=0.001), most advanced ypT stage (P=0.014), and ypN stage (P<0.001) were identified. Pathological complete response (PCR) rates showed statistically significant variations between the three groups (SIPS0: 45.2%, SIPS1: 27.2%, SIPS2: 16.3%, P=0.004). All TRAEs were found in 63.9% (133 cases) of the cases, with serious TRAEs (grade 3-4) accounting for 13.9% (29 cases). TRAEs themselves were not linked with SIPS (P=0.668), while serious TRAEs had a significant correlation with SIPS (P=0.002). Multivariate logistic analysis showed that SIPS2 seemed to confer serious TRAEs [odds radio (OR)=4.044; 95% CI: 1.395-11.722; P=0.010]. For patients classified as SIPS0, 1, or 2, the 3-year DFS was 83.9%, 58.3%, and 39.5% (P<0.001). The 3-year OS for those with SIPS0, 1, or 2 was 88.7%, 72.8%, and 53.5%, respectively (P<0.001). SIPS was substantially correlated with DFS (but not with OS) and could be utilized as an independent predictor [SIPS2: hazard ratio (HR)=3.743, 95% CI: 1.770-7.914, P=0.001; SIPS1: HR=2.303, 95% CI: 1.149-4.616, P=0.019].

**Conclusion:**

The SIPS is associated with serious TRAEs and can be used as a predictor of serious TRAEs in ESCC receiving NICT. SIPS may be employed for pretreatment assessment since it was found to be substantially correlated with DFS.

## Introduction

As one of the most common cancers in China, esophageal cancer (EC), primary esophageal squamous cell carcinoma (ESCC), accounts for more than half the world’s morbidity and mortality (1, 2). Currently, surgical resection after neoadjuvant therapy (NAT) is the main treatment for EC (3). The treatment of EC has been moving toward a comprehensive trend in recent years due to advancements in medicine and treatment techniques, although the therapeutic effect of EC is still unsatisfactory (4). In order to enhance the therapeutic effect of EC, it is crucial to investigate novel therapeutic approaches.

Recently, Immunotherapy, represented by immune checkpoint inhibitors (ICIs), has emerged as a novel therapeutic approach (5). For the treatment of advanced EC, immunotherapy has been advised by current guidelines, and positive outcomes have been reported (6, 7). Based on the excellent efficacy of ICIs in those with advanced disease, an increasing number of clinical trials have examined the safety and efficacy of neoadjuvant immunochemotherapy (NICT) in locally advanced EC (8–10). Currently, as real-world experience with ICIs confirms that some patients can achieve impressive and sustained responses, no accurate predictive indicators are available, and the long-term outcome of NICT is yet unknown. As a result of the widespread use of ICIs and the rise in treatment-related adverse events (TRAEs) due to therapy, there are currently insufficient clinical indicators to predict TRAEs. Therefore, it is of great significance for the prognosis and clinical decision-making of EC to develop indicators that can predict the TRAEs and the long-term outcomes of EC receiving NICT.

According to recent published researches, systemic inflammatory response (SIR) is crucial for the occurrence and progression of malignancies (11, 12). In certain cancers receiving NICT, including those with EC, several inflammatory indicators, reflecting the balance between nonspecific SIR and immunoreaction, such as platelet (PLT) to lymphocyte (LYM) ratio (PLR) and neutrophil (NEU) to LYM ratio (NLR), have been shown to correlate with response and prognosis (13–15). On the other hand, it has also been demonstrated that TRAEs and a number of inflammatory indicators are tightly connected (16, 17). Nonetheless, researches are continuously creating more indicators because of the comparatively low sensitivity and specificity of these indicators for TRAEs and prognostic prediction.

The Scottish inflammatory prognostic score (SIPS), a novel and straightforward inflammatory score that combines albumin (ALB) and NEU, was recently proposed and has shown to be a more accurate predictor in non-small-cell lung cancer (NSCLC) patients receiving first-line ICIs (18). The authors came to the conclusion that SIPS stratifies survival over time periods that are clinically important, which could help patients and clinicians make treatment decisions. In cases of cancer treated with ICIs, the authors support validating the prognostic value of SIPS. Since then, the prognostic value of SIPS has been confirmed in several studies (19–21). However, the study of SIPS in EC has not been reported. Therefore, this study aimed to evaluate the association between TRAEs, prognosis, and SIPS in ESCC receiving NICT.

## Patients and methods

### Study design and patients selection

Analyses were conducted retrospectively on patients with locally advanced ESCC who underwent NICT and radical resection between 2019 and 2020. The following criteria for exclusion were listed: (1) non-radical surgery; (2) diagnosed with non-ESCC; (3) combined with other forms of cancer; (4) in combination with other anticancer therapies; or (5) in combination with other inflammatory, hematologic or autoimmune diseases. In the current study, blood routine and biochemical examinations conducted within a week prior to NICT yielded pretreatment laboratory indices, such as NEU, PLT, LYM, and ALB. The SIPS generates a three-tiered category by combining ALB and NEU (18–21). The PLT to LYM ratio and the NEU to LYM ratio are the definitions of PLR and NLR, respectively (13–15). Systems for TNM staging were carried out using the 8th version (22). The Common Terminology Criteria for Adverse Events, as established by the National Cancer Institute, were used to rate TRAEs (23). The Clavien-Dindo classification was carried out to categorize complications after surgical resection (24). The ethical committee has approved this study and it complies with the Declaration of Helsinki (IRB-2020-183).

### Therapy and follow-up

Two NICT cycles were administered to eligible patients in this investigation, with 200 mg of camrelizumab administered on day 1, albumin-paclitaxel (120 mg/m^2^) administered on days 1 and 8, and carboplatin [5 mg/ml/min on the basis of the area under the curve (AUC)] administered on day 1 of each 21-day cycle. Dose delay was defined as treatments given more than 7 days after the scheduled therapy. The formula utilized to compute the relative dose intensity (RDI) was (delivered dose/standard dose) × 100%. On the basis of the 85% cutoff mark, the patients were then split into two groups (25). McKeown or Ivor Lewis, as a classic surgical procedure, was typically carried out 4-6 weeks after the end of the last cycle (26). There is currently no agreement on adjuvant therapy (AT) in situations where radical surgery after NICT. The evidence-based results reported that immunotherapy, following neoadjuvant chemoradiotherapy (NCRT), significantly increased the duration of disease-free survival (DFS) (27). Immunotherapy was therefore administered as AT in this investigation, though it was optional. Patients received periodic follow-up after the end of their treatment. The final follow-up for this study ended in December 2023.

### Statistical analysis

Chi-square tests or Fisher’s exact tests were used to analyze categorical variables. A one-way ANOVA was used to analyze continuous variables. Based on the analysis of the non-linear relationship between NEU/ALB and DFS/OS, the restricted cubic spline (RCS) was utilized to determine the optimal thresholds for NEU and ALB. Calibration curves, receiver operator characteristic curves (ROCs), decision curve analyses (DCAs), and time-dependent AUCs, were all used to assess the SIPS’s clinical applicability and prognostic value. The predictors in the odds ratio (OR) with 95% confidence interval (CI) for TRAEs were evaluated using logistic analysis. To identify the prognostic factors for DFS/OS, Cox regression analysis in hazard ratio (HR) with 95% CI were also performed. In order to mitigate any explanatory variables that may still be in place and potentially influence the outcomes, we developed three groups (SIPS 0, SIPS 1 and SIPS 2) of patients with balanced covariates using 3-way propensity score matching (PSM) at a ratio of 1:1:1 (28). A logistic regression model was used to determine the propensity score. We assessed covariate balance among the matched cohorts by using standardized mean difference (SMD): values less than 0.10 indicate negligible differences between the matched groups. Using nearest-neighbor matching, a matched cohort with a matching ratio of 1:1:1:1 was produced, with a maximum caliper width of 0.30. The final 3-way matched cohort was expected to include individuals with approximately equal clinical characteristics between the three groups. The duration between enrollment and any of the following events—local, regional, or distant recurrence, or death from any cause—is known as the DFS. While the OS defined as the time from enrollment to death as a result of any cause. All statistical analyses were conducted using SPSS 20.0 and R 4.1.2 software, with statistical significance indicated by P values <0.05.

## Results

### SIPS definition

**Figure 1:**
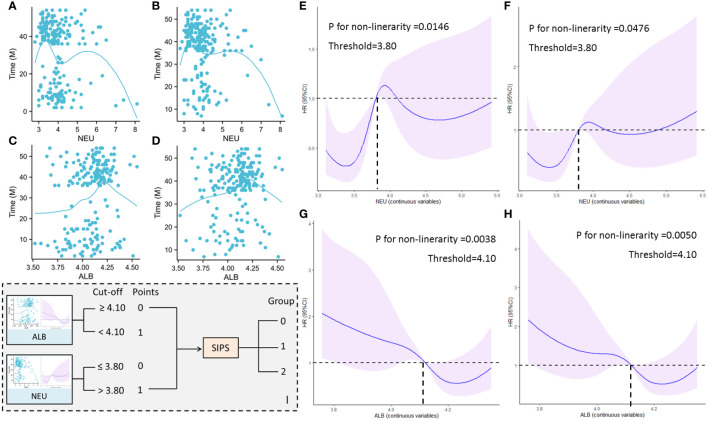
A nonlinear connection between NEU and DFS **(A)** and OS **(B)**. A nonlinear connection between ALB and DFS **(C)** and OS **(D)**. The nonlinear relationship between NEU and DFS **(E)** and OS **(F)** by RCS curves. The nonlinear relationship between ALB and DFS **(G)** and OS **(H)** by RCS curves. The SIPS model composing of ALB and NEU with a three-tier category score **(I)**.

The prognosis of ESCC patients receiving NICT was found to have a nonlinear connection with NEU and ALB in this study (**Figures 1A–D**). Consequently, the nonlinear relationship between NEU/ALB and prognosis (DFS and OS) was examined using RCS curves, and the ideal cut-off values for NEU and ALB were found to be 3.80 and 4.10, respectively (**Figures 1E–H**). The SIPS model composing of ALB and NEU with a three-tier category score (SIPS0, 1, and 2) is then built based on the cut-off values mentioned above (**Figure 1I**).

### Comparison between SIPS and other indices

**Figure 2:**
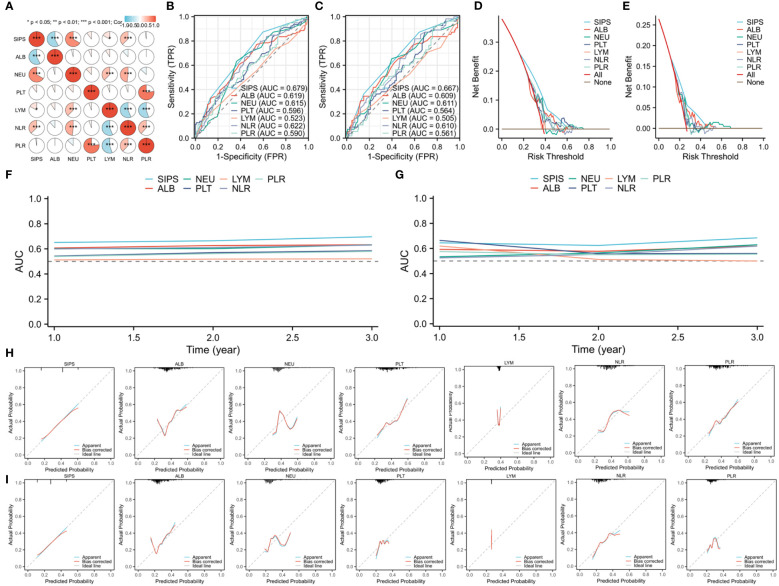
The correlation heatmap between SIPS and additional hematological parameters **(A)**. Comparing SIPS to other indices based on the ROCs in DFS **(B)** and OS **(C)**. Clinical application comparisons based on the DCAs in DFS **(D)** and OS **(E)**. SIPS compared to other indices in the time-dependent AUCs in DFS **(F)** and OS **(G)**. Calibration curves comparisons between SIPS and other indices in DFS **(H)** and OS **(I)**.

**Figure 2A** displays the correlation heatmap between the SIPS and additional hematological parameters. ROCs, DCAs, time-dependent AUCs, and calibration curves between SIPS and other traditional indicators were conducted in order to gain a better understanding of the prognostic usefulness and clinical usability of SIPS. Comparing SIPS to other indices, the ROC curves showed that it had the biggest AUC (DFS=0.679 and OS=0.667), suggesting a stronger predictive ability (**Figures 2B, C**). Additionally, the DCA curves supported the superior clinical application of SIPS in DFS and OS when compared to other indices (**Figures 2D, E**). In the time-dependent AUC curves, SIPS again had the superior predictive value when compared to other indices (**Figures 2F, G**). Finally, compared to other indices, the calibration curves also demonstrated that SIPS achieves a high enough degree of calibration (**Figures 2H, I**).

### Characteristics grouped by SIPS

The patient characteristics are shown in **Table 1**. A total of 208 ESCC patients were included in this study. The patients were divided into three groups according to SIPS, including 62 cases of SIPS0, 103 cases of SIPS1 and 43 cases of SIPS2. Among patients with SIPS2, the oldest age (P=0.006), lowest BMI (P=0.001), longest tumor length (P=0.001), most advanced ypT (P=0.014) and ypN stage (P<0.001) were identified. Pathological complete response (PCR) rates showed statistically significant variations between the three groups (SIPS0: 45.2%, SIPS1: 27.2%, SIPS2: 16.3%, P=0.004). In addition, patients with SIPS2 had the longest stay after surgery (P=0.047) and largest number of positive lymph nodes (P=0.001). Regarding to postoperative complications, the SIPS was significantly associated with major complications (P=0.032), especially for respiratory complications (P=0.035).

**Table 1:** Patient characteristics grouped by SIPS in ESCC receiving NICT.

	SIPS 0 (n=62)	SIPS 1 (n=103)	SIPS 2 (n=43)	P-value
Clinical characteristics				
Sex (female/male, n)	8/54	11/92	5/38	0.911
Age (mean ± SD, years)	60.4 ± 7.6	63.2 ± 7.5	64.7 ± 5.5	0.006
Smoking history (yes/no, n)	44/18	74/29	31/12	0.990
Drinking history (yes/no, n)	46/16	70/33	31/12	0.678
BMI (mean ± SD, Kg/m^2^)	22.2 ± 1.86	21.4 ± 1.97	20.9 ± 1.52	0.001
Tumor location (U/M/L, n)	7/37/18	8/61/34	5/23/15	0.869
Differentiation (W/M/P, n)	18/25/19	20/46/37	9/19/15	0.708
Vessel invasion (yes/no, n)	7/55	15/88	7/36	0.744
Perineural invasion (yes/no, n)	7/55	17/86	12/31	0.082
Tumor length (mean ± SD, cm)	1.38 ± 1.58	2.09 ± 1.81	2.69 ± 1.82	0.001
cTNM stage (II/III/IVa, n)	14/40/8	20/66/17	10/22/11	0.451
ypT stage (T0/T1-2/T3-4a, n)	28/17/17	28/40/35	7/15/21	0.014
ypN stage (N0/N1-3, n)	49/13	58/45	18/25	<0.001
PCR (yes/no, n)	28/34	28/75	7/36	0.004
Dose relative intensity				
Dose reduction (yes/no, n)	24/38	55/48	21/22	0.187
Dose delay (yes/no, n)	12/50	26/77	14/29	0.306
RDI (<85%/≥85%, n)	4/58	25/78	13/30	0.004
Treatment-relative adverse effects				
Any TRAEs (yes/no, n)	39/23	64/39	30/13	0.668
Serious TRAEs (yes/no, n)	6/56	10/93	13/30	0.002
Capillary proliferation (yes/no, n)	19/43	36/67	17/26	0.639
Hypocytosis (yes/no, n)	10/52	25/78	17/26	0.024
Neutropenia (yes/no, n)	6/56	17/86	13/30	0.023
Leukopenia (yes/no, n)	8/54	17/86	15/28	0.012
Thrombocytopenia (yes/no, n)	5/57	12/91	8/35	0.260
Anemia (yes/no, n)	8/54	20/83	18/25	0.001
Hypothyroidism (yes/no, n)	8/54	12/91	7/36	0.750
Decreased appetite (yes/no, n)	12/50	24/79	7/36	0.605
Nausea or vomiting (yes/no, n)	9/53	15/88	8/35	0.806
Asthenia or fatigue (yes/no, n)	10/52	23/80	8/35	0.612
Alopecia (yes/no, n)	20/42	31/72	14/29	0.938
Diarrhea (yes/no, n)	5/57	14/89	8/35	0.277
Constipation (yes/no, n)	5/57	10/3	9/34	0.119
Fever (yes/no, n)	3/59	6/96	5/38	0.391
Rash (yes/no, n)	2/60	5/97	3/40	0.380
Neurotoxic effects (yes/no, n)	1/61	3/100	5/40	0.151
Abnormal hepatorenal function (yes/no, n)	16/46	22/81	14/29	0.357
Immune-related adverse effects (yes/no, n)	4/58	7/96	6/37	0.342
Intraoperative characteristics				
Operative time (mean ± SD, min)	214.8 ± 27.0	222.8 ± 32.3	226.2 ± 46.8	0.195
Operative blood loss (mean ± SD, ml)	148.7 ± 70.1	143.4 ± 52.1	161.4 ± 82.4	0.313
Stay after surgery (mean ± SD, day)	12.5 ± 3.9	13.8 ± 7.1	15.9 ± 9.2	0.047
Total LNs (mean ± SD, n)	22.3 ± 8.1	22.1 ± 9.8	22.1 ± 8.5	0.981
Positive LNs (mean ± SD, n)	0.50 ± 1.20	1.25 ± 2.09	1.98 ± 2.60	0.001
Negative LNs (mean ± SD, n)	21.8 ± 7.8	20.8 ± 9.9	20.0 ± 7.9	0.592
Postoperative complications				
Major morbidity (yes/no, n)	17/45	29/74	21/22	0.032
Respiratory complications (yes/no, n)	8/54	18/85	14/29	0.035
Anastomotic leakage (yes/no, n)	5/57	10/93	6/37	0.62
Chylothorax (yes/no, n)	1/61	1/102	2/41	0.329
Wound infection (yes/no, n)	2/60	5/98	5/38	0.205
Recurrent laryngeal nerve injury (yes/no, n)	5/56	12/91	7/36	0.585
Hematological indices				
ALB (mean ± SD, g/dl)	4.19 ± 0.08	4.09 ± 0.19	3.91 ± 0.14	<0.001
NEU (mean ± SD, 10^9^/L)	3.42 ± 0.28	4.00 ± 0.70	4.65 ± 0.95	<0.001
LYM (mean ± SD, 10^9^/L)	1.40 ± 0.25	1.43 ± 0.24	1.52 ± 0.23	0.029
PLT (mean ± SD, 10^9^/L)	191.6 ± 39.2	195.9 ± 49.1	208.3 ± 50.5	0.186
NLR (mean ± SD)	2.52 ± 0.47	2.86 ± 0.53	3.08 ± 0.54	<0.001
PLR (mean ± SD)	140.9 ± 35.3	141.5 ± 43.8	138.9 ± 39.6	0.937

SIPS, Scottish inflammatory prognostic score; ESCC, esophageal squamous cell carcinoma; NICT, neoadjuvant immunochemotherapy; SD, standard deviation; BMI, body mass index; U/M/L, upper/middle/lower; W/M/P, well/moderate/poor; TNM, tumor node metastasis; PCR, pathological complete response; TRAEs, treatment-related adverse effects; RDI, relative dose intensity; LNs, lymph nodes; ALB, albumin; NEU, neutrophil; LYM, lymphocyte; PLT, platelet; NLR, neutrophil to lymphocyte ratio; PLR, platelet to lymphocyte ratio.

### SIPS and TRAEs

**Figure 3:**
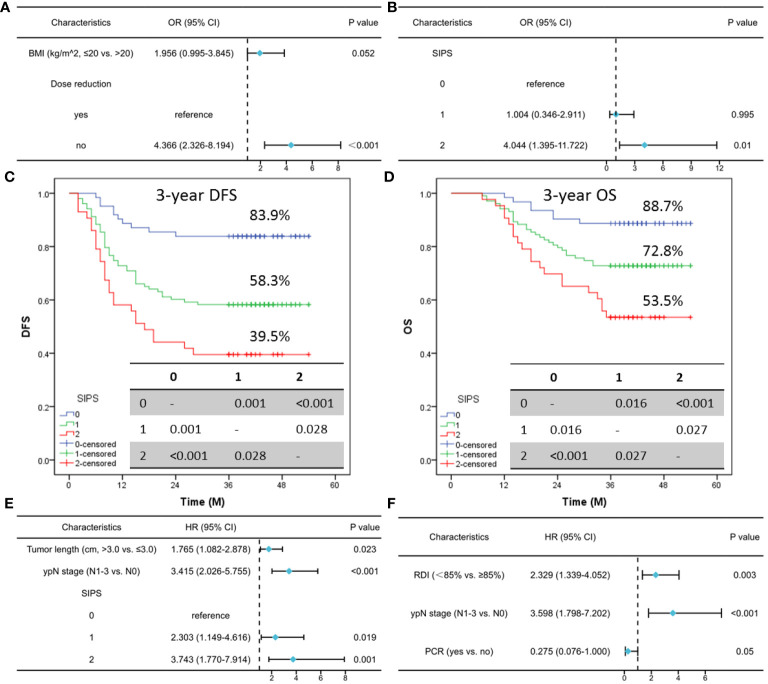
Multivariate logistic analysis of risk factors for TRAEs **(A)** and serious TRAEs **(B)**. For patients classified as SIPS0, SIPS1, or SIPS2, the 3-year DFS was 83.9%, 58.3%, and 39.5% **(C)**. The 3-year OS for those with SIPS0, SIPS1, or SIPS2 was 88.7%, 72.8%, and 53.5%, respectively **(D)**. Multivariate Cox analysis in DFS **(E)** and OS **(F)**.

All TRAEs were found in 63.9% (133 cases) of the cases in the current study, with serious TRAEs (grade 3-4) accounting for 13.9% (29 cases). As shown in **Table 1**, TRAEs themselves were not linked with SIPS (P=0.668), while serious TRAEs had a significant correlation with SIPS (P=0.002). Furthermore, an analysis of the correlation between SIPS and TRAEs revealed statistically differences in the hypocytosis (P=0.024), especially in the neutropenia (P=0.023), leukopenia (P=0.012), and anemia (P=0.001). In order to gain a deeper understanding of the relationship between TRAEs and SIPS, additionally, a trajectory analysis was conducted to explore the correlation between TRAEs and SIPS at each treatment cycle, as well as the association between TRAEs and SIPS at different grades. The results in the study indicated that SIPS was associated with hematological TRAEs, especially in the first cycle (**Supplementary Table S1**). Finally, multivariate logistic analysis demonstrated that SIPS2 (OR=4.044; 95% CI: 1.395-11.722; P=0.010) seemed to confer serious TRAEs, while not for SIPS1 (OR=1.004; 95% CI: 0.346-2.911; P=0.995) (**Figures 3A, B**).

### SIPS and survival

Seventy-nine cases in all had relapsed: 32 (40.5%) patients had a local recurrence and 47 (59.5%) patients had a distant recurrence. Of the 79 patients who relapsed, 55 cases died. For patients classified as SIPS0, SIPS1, or SIPS2, the 3-year DFS was 83.9%, 58.3%, and 39.5% (P<0.001, **Figure 3C**). The 3-year OS for those with SIPS0, SIPS1, or SIPS2 was 88.7%, 72.8%, and 53.5%, respectively (P<0.001, **Figure 3D**). Significant prognostic factors in the univariate analyses for DFS and OS were then recruited for further analyses (**Supplementary Table S2**). Multivariate Cox analyses revealed that SIPS was substantially correlated with DFS (but not with OS) and could be utilized as a novel independent predictor (SIPS2: HR=3.743, 95% CI: 1.770-7.914, P=0.001; SIPS1: HR=2.303, 95% CI: 1.149-4.616, P=0.019) (**Figures 3E, F**). Regarding clinical characteristics, there were significant differences across the SIPS groups. Therefore, a 1:1:1 PSM result is shown in **Supplementary Table S3**. In the current investigation, if the PSM is used for forced matching, the final three groups consist of 25 patients each. The results also indicated that SIPS had a significant relationship with 3-year DFS (P=0.025) but not with 3-year OS. As an independent prognostic factor for ESCC receiving NICT, SIPS is associated with DFS (but not with OS) (**Supplementary Figure S1**).

## Discussion

The predictive usefulness of SIPS in ESCC undergoing NICT was first verified in the current study. Patients with SIPS2 had the worst 3-year DFS and OS. The findings suggested that SIPS might offer practical predictive data to support patients and clinicians in making treatment decisions. Additionally, TRAEs were not associated with SIPS, but there was an association between SIPS and serious TRAEs. There were statistical differences between the SIPS and hematological TRAEs. Multivariate logistic analysis showed that SIPS2 seemed to confer serious TRAEs, while this was not seen for SIPS1. Therefore, we concluded that the SIPS is associated with serious TRAEs and can be used as a predictor of serious TRAEs in ESCC receiving NICT. A substantial proportion of our findings align with previously results (20), indicating that SIPS, upon further validation, may be utilized in the future as a cheap and accessible potential indicator to forecast the emergence of TRAEs and prognosis.

It has been demonstrated that the SIPS, which was firstly proposed, is a more accurate predictor in NSCLC patients receiving first-line ICIs (18). The authors concluded that SIPS was predictive of both progression-free survival (PFS) and OS and would aid patients and doctors in making treatment decisions. Aiming to investigate the prognostic significance of SIPS after failure of first-line pembrolizumab for NSCLC to support subsequent management decisions, another study included 211 patients for advanced NSCLC was proposed (19). SIPS was found to be predictive of post-progression overall survival (ppOS) on multivariate analysis, with ppOS being stratified (SIPS2: 0.8 months; SIPS1: 1.8 months; SIPS0: 8.1 months). The authors also deduced that SIPS would be useful in identifying a select subset of patients who stand to gain the most from further therapeutic approaches. Another study aimed to explore the relationship between SIPS, TRAEs, and survival outcomes in 262 NSCLC patients (20). The results also demonstrated that SIPS predicted PFS and OS. Furthermore, in patients whose primary malignancy was unknown, SIPS was an independent predictor of survival (21). The authors came to the additional conclusion that SIPS successfully used clinicopathological variables to stratify survival in patients with both favorable-risk and poor-risk patients.

The use of immune checkpoint inhibitors (ICIs) has increased during the past decade for a variety of cancer indications. TRAEs from ICIs are a major cause of morbidity and, in certain cases, can result in treatment-related death (29, 30). Recently, a meta-analysis reported that more than 300 different types of TRAEs were reported in 125 studies. Overall, out of 18610 patients in 106 studies, 12277 (66.0%) developed at least one TRAE of any grade, and out of 18715 individuals in 110 studies, 2627 (14.0%) developed at least one serious TRAE (31). The statistics from this study are essentially the same as the data from the previous study (63.9% of the cases in the current study had any TRAEs, and 13.9% had serious TRAEs). Considerable prospects exist for enhancing the identification of individuals who are more susceptible to treatment-related toxicity, precisely diagnosing TRAEs at an earlier stage of the condition, and creating more customized treatment plans when issues emerge (32).

Recent studies have revealed that SIR was associated with higher risk of developing TRAEs in retrospective series (16, 17, 20). A study aimed to explore the relationship between SIPS, TRAEs, and survival outcomes in 262 NSCLC patients. Based on the data, the SIPS was able to predict that all patients will experience any TRAEs (P=0.011) (20). In a retrospective review of advanced NSCLC patients treated with immunotherapy, NLR and PLR were frequently linked to TRAEs, and multivariate analyses validated PLR as an independent prognostic index of TRAEs (33). In the present study, there was no significant correlation found between SIPS and TRAEs (P=0.668), while SIPS and serious TRAEs were significantly correlated (P=0.002). Furthermore, statistically significant differences in the hematological TRAEs were found by analyzing the link between the SIPS and a couple of these TRAEs. Finally, SIPS2 (OR=4.044; 95% CI: 1.395-11.722; P=0.010) seemed to confer serious TRAEs, while this was not seen for SIPS1 (OR=1.004; 95% CI: 0.346-2.911; P=0.995).

Our study has several limitations, including its retrospective nature, single-institutional design, and limited sample size. Additionally, because our analysis was retrospective, bias in the documentation of TRAE incidence and risk variables may exist, and some TRAEs may not have been thoroughly documented. Furthermore, even with our stringent exclusion criteria, SIPS, derived from peripheral blood, might still be impacted by other confounding variables. In addition, the current study is the lack of an external validation cohort. For this and other patient groups receiving NICT, we heartily recommend additional research that includes independent validation of SIPS. Finally, PSM has become a method most frequently employed in clinical work to analyze observational data. However, significant or even enormous sample sizes were used in simulation experiments to essentially examine the PSM’s performance. Therefore, choosing this approach could present certain efficiency challenges when the sample size is small (34). Furthermore, a debate concerning the appropriateness of PSM was prompted by certain recent research that expressed issues regarding its use, particularly in light of the increasing postmatching covariate imbalance (35). Although SIPS is associated with DFS in PSM results in the present study, the small number of enrolled samples (25 cases) may also lead to increased statistical bias. Therefore, more large sample, randomized, prospective clinical studies are needed to validate the predictions of SIPS in prognosis and TRAEs.

## Conclusion

In conclusion, SIPS is associated with serious TRAEs and can be used as a predictor of serious TRAEs in ESCC receiving NICT. SIPS may be employed for pretreatment assessment since it was found to be substantially correlated with DFS.

## Data availability statement

The original contributions presented in the study are included in the article/**Supplementary Material**. Further inquiries can be directed to the corresponding authors.

## Ethics statement

The ethical committee has approved this study and it complies with the Declaration of Helsinki (IRB-2020-183). The studies were conducted in accordance with the local legislation and institutional requirements. The ethics committee/institutional review board waived the requirement of written informed consent for participation from the participants or the participants’ legal guardians/next of kin. Since the study was retrospective and all data was anonymous, informed consent was waived.

## Author contributions

QZ: Investigation, Methodology, Writing – original draft, Writing – review & editing. LW: Data curation, Formal analysis, Writing – review & editing. XY: Investigation, Methodology, Writing – review & editing. JF: Conceptualization, Project administration, Writing – original draft, Writing – review & editing. QC: Conceptualization, Project administration, Writing – original draft, Writing – review & editing.

## Supplementary material

Supplementary Figure 1 For patients classified as SIPS0, SIPS1, or SIPS2, the 3-year DFS after PSM was 88.0%, 60.0%, and 52.0% **(A)**. The 3-year OS after PSM for those with SIPS0, SIPS1, or SIPS2 was 88.0%, 76.0%, and 72.0%, respectively **(B)**. Multivariate Cox analysis in DFS **(C)** and OS **(D)** after PSM.
