# Supplementary figures and images for: Association of the Scottish inflammatory prognostic score with treatment-related adverse events and prognosis in esophageal cancer receiving neoadjuvant immunochemotherapy
Source: Front Immunol. 2024 Jul 5;15:1418286. doi: 10.3389/fimmu.2024.1418286 (PMC11257864; doi:10.3389/fimmu.2024.1418286)

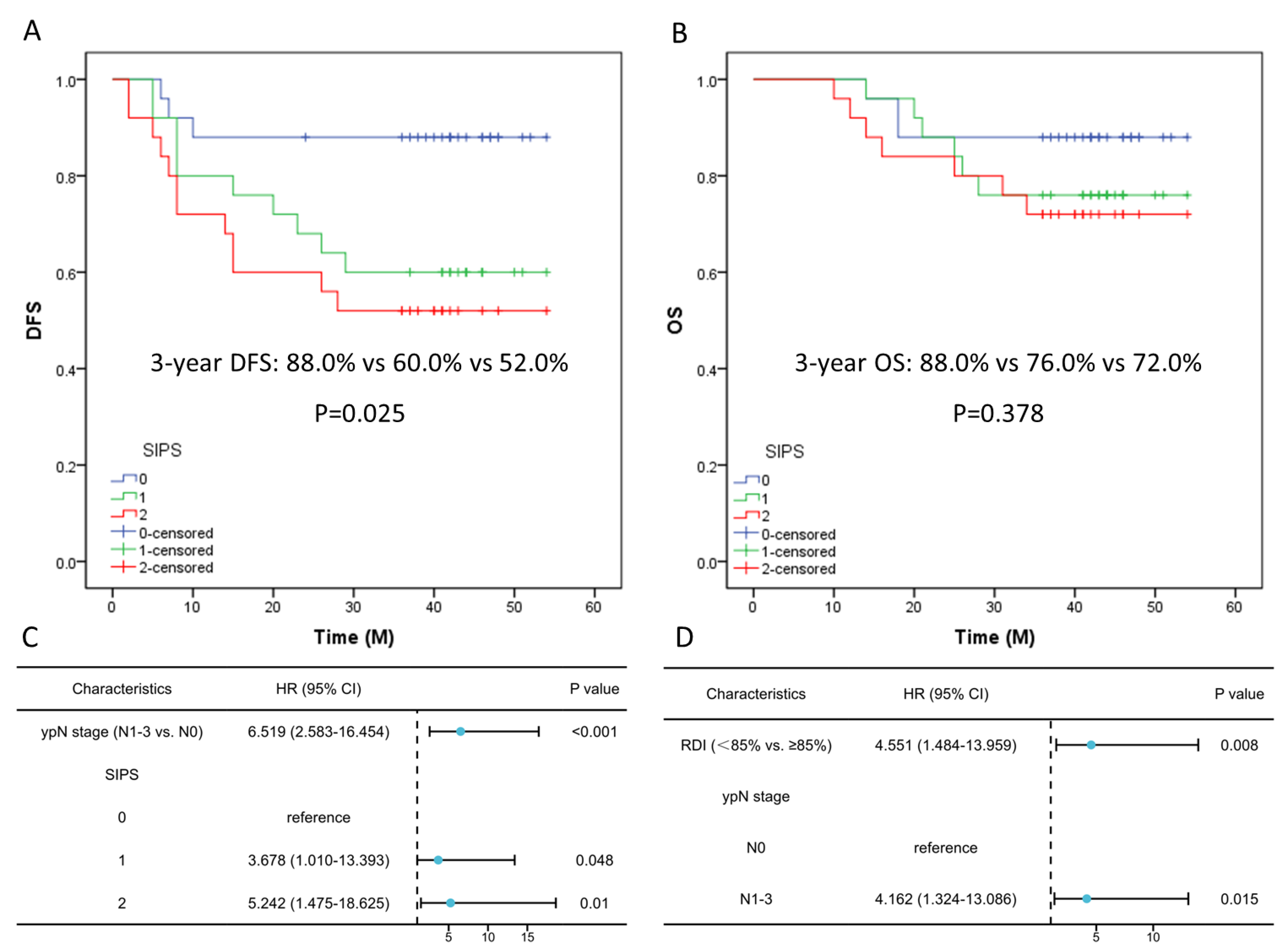

Supplement: Supplementary Figure 1 — For patients classified as SIPS0, SIPS1, or SIPS2, the 3-year DFS after PSM was 88.0%, 60.0%, and 52.0% (A). The 3-year OS after PSM for those with SIPS0, SIPS1, or SIPS2 was 88.0%, 76.0%, and 72.0%, respectively (B). Multivariate Cox analysis in DFS (C) and OS (D) after PSM. [file Image_1.jpeg]
