# Supplementary material for: Association of the Scottish inflammatory prognostic score with treatment-related adverse events and prognosis in esophageal cancer receiving neoadjuvant immunochemotherapy
Source: Front Immunol. 2024 Jul 5;15:1418286. doi: 10.3389/fimmu.2024.1418286 (PMC11257864; doi:10.3389/fimmu.2024.1418286)
Supplement: Supplementary file 2 [file Table_1.docx]

**Table S1 Trajectory analysis of the TRAEs at each treatment cycle in ESCC receiving NICT**

|  | Cycle 1  Grade 1-2 Grade 3-4  0 1 2 P 0 1 2 P |  |  | Cycle 2  Grade 1-2 Grade 3-4  0 1 2 P 0 1 2 P |
| --- | --- | --- | --- | --- |
| Any TRAEs (n)  Capillary proliferation (n)  Hypocytosis (n)  Neutropenia (n)  Leukopenia (n)  Thrombocytopenia (n)  Anemia (n)  Hypothyroidism (n)  Decreased appetite (n)  Nausea or vomiting (n)  Asthenia or fatigue (n)  Alopecia (n)  Diarrhea (n)  Constipation (n)  Fever (n)  Rash (n)  Neurotoxic effects (n)  Abnormal hepatorenal function (n)  Immune-related adverse effects (n) | 28 50 17 0.606 5 8 10 0.030  15 28 14 0.638 0 0 0 -  5 22 11 0.038 2 3 6 0.028  3 10 7 0.148 1 5 6 0.034  4 10 7 0.272 2 6 8 0.006  2 5 5 0.085 2 6 3 0.379  6 16 14 0.008 1 2 4 0.048  5 10 6 0.620 0 0 0 -  10 22 7 0.634 0 0 0 -  6 14 6 0.724 0 0 0 -  8 16 6 0.893 0 0 0 -  18 28 12 0.968 0 0 0 -  4 12 7 0.267 0 1 0 0.897  5 9 6 0.576 0 0 0 -  3 4 3 0.387 0 0 0 -  2 4 3 0.379 0 0 0 -  1 2 3 0.135 0 0 0 -  12 18 10 0.721 0 0 0 -  2 4 3 0.379 0 0 1 0.121 |  |  | 20 42 15 0.519 3 4 6 0.089  17 32 13 0.882 0 0 0 -  8 20 12 0.158 2 4 5 0.079  3 10 6 0.255 3 4 5 0.224  5 11 8 0.259 2 3 5 0.105  3 4 4 0.447 1 3 4 0.147  5 13 11 0.034 2 2 5 0.065  4 7 5 0.582 0 1 0 0.897  12 22 7 0.778 0 0 0 -  9 15 8 0.806 0 0 0 -  10 23 7 0.532 0 0 0 -  20 31 14 0.938 0 0 0 -  5 8 6 0.501 0 0 0 -  4 7 8 0.082 0 0 0 -  2 5 4 0.409 0 0 0 -  2 5 3 0.380 0 0 0 -  1 3 3 0.151 0 0 0 -  14 20 12 0.527 1 0 1 0.854  3 6 5 0.403 0 1 1 0.234 |

**Abbreviations:** TRAEs: treatment-related adverse effects; SIPS: Scottish inflammatory prognostic score; ESCC: esophageal squamous cell carcinoma; NICT: neoadjuvant immunochemotherapy.
