# Supplementary material for: Association of the Scottish inflammatory prognostic score with treatment-related adverse events and prognosis in esophageal cancer receiving neoadjuvant immunochemotherapy
Source: Front Immunol. 2024 Jul 5;15:1418286. doi: 10.3389/fimmu.2024.1418286 (PMC11257864; doi:10.3389/fimmu.2024.1418286)
Supplement: Supplementary file 3 [file Table_2.docx]

**Table S2 Univariate Cox analyses of prognostic factors in DFS and OS for ESCC receiving NICT**

|  | DFS  HR (95% CI) P-value | OS  HR (95% CI) P-value |
| --- | --- | --- |
| Sex (male vs. female)  Age (years, >70 vs. ≤70)  BMI (Kg/m^2^, >20 vs. ≤20)  Smoking history (yes vs. no)  Drinking history (yes vs. no)  Dose reduction (yes vs. no)  Dose delay (yes vs. no)  RDI (<85% vs. ≥85%)  TRAEs (yes vs. no)  Tumor location  upper  middle  lower  Differentiation  well  moderate  poor  Vessel invasion (yes vs. no)  Perineural invasion (yes vs. no)  Tumor length (cm, >3 vs. ≤3)  ypT stage  T0  T1-2  T3-4a  ypN stage (N1-3 vs. N0)  PCR (yes vs. no)  Adjuvant therapy (yes vs. no) | 0.801 (0.423-1.515) 0.495  0.882 (0.509-1.526) 0.653  0.444 (0.283-0.697) <0.001  0.812 (0.507-1.298) 0.383  1.180 (0.722-1.930) 0.509  1.187 (0.764-1.846) 0.446  1.223 (0.742-2.015) 0.430  2.184 (1.343-3.553) 0.002  0.633 (0.407-0.986) 0.043  reference  0.464 (0.237-0.908) 0.025  0.729 (0.265-1.456) 0.371  reference  1.526 (0.786-2.964) 0.212  2.266 (1.175-4.368) 0.015  2.469 (1.455-4.187) 0.001  2.122 (1.276-3.528) 0.004  2.919 (1.852-4.599) <0.001  reference  2.708 (1.311-5.597) 0.007  4.944 (2.478-9.866) <0.001  5.024 (3.103-8.135) <0.001  0.268 (0.138-0.521) <0.001  1.439 (0.873-2.371) 0.153 | 0.894 (0.404-1.975) 0.781  0.578 (0.273-1.224) 0.152  0.426 (0.249-0.728) 0.002  1.194 (0.651-2.191) 0.566  1.590 (0.838-3.015) 0.156  1.317 (0.775-2.236) 0.308  1.542 (0.878-2.708) 0.132  2.812 (1.622-4.875) <0.001  0.636 (0.374-1.081) 0.094  reference  0.399 (0.187-0.853) 0.018  0.562 (0.256-1.234) 0.151  reference  2.241 (0.916-5.484) 0.077  3.076 (1.261-7.499) 0.013  3.207 (1.790-5.746) <0.001  3.207 (1.838-5.596) <0.001  3.475 (2.041-5.917) <0.001  reference  6.209 (1.837-20.984) 0.003  12.16 (3.728-39.681) <0.001  6.227 (3.338-11.616) <0.001  0.111 (0.035-0.356) <0.001  1.076 (0.567-2.040) 0.823 |
| SIPS  0  1  2 | reference  3.063 (1.539-6.098) 0.001  5.219 (2.513-10.836) <0.001 | reference  2.664 (1.163-6.099) 0.020  5.013 (2.119-11.862) <0.001 |

**Abbreviation:** SIPS: Scottish inflammatory prognostic score; ESCC: esophageal squamous cell carcinoma; BMI: body mass index; PCR: pathological complete response; HR: hazard ratio; CI: confidence interval; TNM: tumor node metastasis; DFS: disease-free survival; OS: overall survival.
