# Supplementary material for: Association of the Scottish inflammatory prognostic score with treatment-related adverse events and prognosis in esophageal cancer receiving neoadjuvant immunochemotherapy
Source: Front Immunol. 2024 Jul 5;15:1418286. doi: 10.3389/fimmu.2024.1418286 (PMC11257864; doi:10.3389/fimmu.2024.1418286)
Supplement: Supplementary file 4 [file Table_3.docx]

**Table S3 Clinical Characteristics before and after PSM in ESCC receiving NICT**

|  | Before PSM  SIPS0(n=62) SIPS1(n=103) SIPS2(n=43) P-value |  |  | After PSM  SIPS0(n=25) SIPS1(n=25) SIPS2(n=25) P-value |
| --- | --- | --- | --- | --- |
| Sex (male/female, n)  Age (years, mean ± SD)  BMI (Kg/m^2^, mean ± SD)  Smoking history (yes/no, n)  Drinking history (yes/no, n)  Tumor location (U/M/L, n)  Differentiation (W/M/P, n)  Vessel invasion (yes/no, n)  Perineural invasion (yes/no, n)  Tumor length (cm, mean ± SD)  cTNM (II/III/IVA, n)  ypT stage (T0/T1-2/T3-4a, n)  ypN stage (N0/N1-3, n)  PCR (yes/no, n)  Dose reduction (yes/no, n)  Dose delay (yes/no, n)  RDI (<85%/≥85%, n)  TRAEs (yes/no, n) | 8/54 11/92 5/38 0.911  60.4 ± 7.6 63.2 ± 7.5 64.7 ± 5.5 0.006  22.2 ± 1.86 21.4 ± 1.97 20.9 ± 1.52 0.001  44/18 74/29 31/12 0.990  46/16 70/33 31/12 0.678  7/37/18 8/61/34 5/23/15 0.869  18/25/19 20/46/37 9/19/15 0.708  7/55 15/88 7/36 0.744  7/55 17/86 12/31 0.082  1.38 ± 1.58 2.09 ± 1.81 2.69 ± 1.82 0.001  14/40/8 20/66/17 10/22/11 0.451  28/17/17 28/40/35 7/15/21 0.014  49/13 58/45 18/25 <0.001  28/34 28/75 7/36 0.004  24/38 55/48 21/22 0.178  12/50 26/77 14/29 0.306  4/58 25/78 13/30 0.004  39/23 64/39 30/13 0.668 |  |  | 3/22 1/24 3/22 1.000  63.8 ± 7.5 62.9 ± 7.4 63.7 ± 4.7 0.872  21.2 ± 1.74 21.3 ± 2.06 21.4 ± 1.50 0.923  7/18 4/21 4/21 0.472  6/19 8/17 7/18 0.820  3/16/6 1/17/7 2/14/9 0.326  7/12/6 5/11/9 5/11/9 0.865  3/22 2/23 3/22 1.000  3/22 3/22 4/21 0.679  2.08 ± 1.68 1.84 ± 1.91 1.84 ± 1.52 0.849  7/12/6 8/15/2 6/15/4 0.830  7/7/11 9/7/9 7/9/9 0.914  15/10 15/10 15/10 1.000  7/18 9/16 9/16 0.778  10/15 11/14 12/13 0.850  4/21 4/21 7/18 0.472  3/22 3/22 4/21 0.679  18/7 15/10 20/5 0.295 |

**Abbreviations:** ESCC: esophageal squamous cell carcinoma; NICT: neoadjuvant immunochemotherapy; SIPS: Scottish inflammatory prognostic score; SD: standard deviation; BMI: body mass index; U/M/L: upper/middle/lower; W/M/P: well/moderate/poor; TNM: tumor node metastasis; PCR: pathological complete response; RDI: relative dose intensity; TRAEs: treatment-related adverse effects.
